# Lipid Disorders in Elderly Hypertensive Patients

**DOI:** 10.1155/2012/684515

**Published:** 2011-12-29

**Authors:** Blas Gil-Extremera

**Affiliations:** Departamento de Medicina, Facultad de Medicina, Universidad de Granada, Avdenida Madrid 11, 18012 Granada, Spain

## Abstract

Lipid disorders are a common clinical challenge in the western countries. In patients with dyslipemia (total cholesterol > 200 mg/dl, HDL cholesterol < 35 mg/dl, LDL cholesterol > 130 mg/dl and triglycerides > 150 mg/dl) it is mandatory to normalize blood pressure (<130/80 mmHg) as well to reduce LDL-C values to normal levels by using drugs to inhibit of endogenous and exogenous cholesterol, to decrease triglycerides, and increases HDL-C up to normal range. It is also essential to maintain for this purpose suitable dietetic measures (reduction of unsatured fats and salt intakes—<2.5 g/daily) and without interruption, to support pharmacologic treatment in most of the patients.

## 1. Introduction

During many years I have been observing very often some misunderstanding therapeutic ideas from several physicians about the management of patients with lipid disorders mostly in older hypertensive patients. This situation is directly related to an insufficient therapeutic control due to inadequate drug dosages. However, we must not neglected this point because good control is crucial for clinical point of view and for the prognosis of the patients.

It is well established the relevance to provide some measures for the prevention and delay of atherosclerosis that is closely related to the well-known cardiovascular risk factors, such as, hypertension, smoking habit, dyslipemia, obesity, diabetes, and many others disorders. On the other hand, severe or fatal cardiovascular events related to the risk factors are also important in order to offer the patients a better management to reduce stroke, artery coronary disease, peripheral arteriopathy, and other vascular diseases in western countries.

Hypertension, together with smoking habit and high blood lipid levels, represents the most important pathogenic cause for atherosclerosis and subsequent clinical severe diseases.

The following ideas are based on my clinical experience during many years on this particular clinical disorder.

## 2. Lowering Lipid Levels

As part of an overall strategy to stop atherosclerosis, control of hypertension is very important (BP ≤ 140/80 mmHg) in all cases using general measures (diet, salt restriction, relief of stress, regular aerobic exercise, and dietary management: caloric restriction, reduction of cholesterol and saturated fats intake) as well as drug therapy when necessary [1]. Smoking habit, for example, must be stopped as soon as possible, and Current National Guidelines recommend having cholesterol level screening in adults [2]. They also propose to obtain a fasting lipid profile (including total cholesterol, triglycerides, LDL cholesterol, and HDL cholesterol) in patients with known vascular diseases and those with several risk factors or elevated total cholesterol levels.

## 3. Some Frequent Errors Observed in the Clinical Practice

Unfortunately, three types of mistakes are very common present in clinical practice by many physicians: (1) some colleagues take as normal lipid values clearly high and neglecting the reference values expressed on Table 1. At this point, many patients with moderately high cholesterol, triglycerides, LDL-cholesterol and decreased HDL cholesterol with values below ranging between 35 and 40 mg/dL do not receive any treatment; (2) it is well-known that statin/fibrate combination therapy is not recommended in same patient to treat mixed dyslipemia; but unfortunaly, this procedure is maintain by physicians in many cases. These associations are not adequate because of their increasing side effects rhabdomyolysis, severe and even fatal in some patients; (3) in my clinical experience, the most common error that I have observed is the discontinuation of lipid lowering therapy when normal values are reached; obviously expected, lipid values become again pathologically high after suspension of therapy (statins, fibrates, or ezetimibe) (Table 2).

Nevertheles, thanks to the therapeutic arsenal available today, observed normalization of lipid values in a large percentage of patients. The 4S study [3] demonstrated for the first time that intensive treatment to decrease lipid levels achieved reduced mortality rate in patients with coronary heart disease and cholesterol values between 212 and 309 mg/dL (mean: 260 mg/dL). The CARE study [4] showed beneficial effects in patients with history of coronary heart disease and total cholesterol of 175 to 240 mg/dL (mean: 211 mg/dL); the LIPID trial [5] showed an improvement in total cardiovascular mortality rate with cholesterolemia levels below those reported in the 4S study (mean: 220 mg/dL).

The AFCAPS/TexCAPS study [6] showed the benefit of lipid-lowering treatment of cardiovascular morbidity and mortality rate in primary prevention in individuals with levels between 180 and 264 mg/dL (mean, 221 mg/dL) aged 45–75 years, as well as a slight decrease of HDL-C levels in individuals with cholesterol values considered within the normal limits (180–264 mg/dL; mean: 221 mg/dL). The PROVE-IT [7] demonstrated that intensive lipid-lowering therapy versus standard treatment reduced significantly cardiovascular morbidity and mortality rate after acute coronary syndromes.

These studies have cleared the way to recommend statins therapy in patients treated for secondary prevention (high risk) and primary prevention with other related risk factors (Table 3).

Our experience demonstrated in hypertensive patients older than 60 as the coadministration of ezetimibe/simvastatin + fenofibrate improved atherogenic lipid profile with mixed hyperlipidemia [8]. Another recent study, with participation of my group [9] showed that the association of “extended-released niacin and laropiprant (ERN/LRDT) + statin” significantly improved the lipid profile compared to *the run-in dose doubled,* and it was generally well tolerated in patients with primary hypercholesterolemia and mixed dyslipemia. The protocol was the following: after a 2- to 6-week run-in statin (simvastatin 10 or 20 mg or atorvastatin 10 mg) period, 1216 patients were randomised equally to one of two treatment groups in a double-blind fashion: group 1 received ERN/LRPT (1 g) plus the run-in statin dose and advanced to ERN/LRPT (2 g) after 4 weeks for an additional 8 weeks, with no adjustments to the run-in statin dose; group 2 received simvastatin or atorvastatin at twice their run-in statin dose and remained on this stable dose for 12 weeks.

Some of the molecules under study (clinical trials phase II or III) in which our unit is participating actively are expressed in Merck Pipeline: cardiovascular: MK-0736, MK 6621 (vernakalant), atherosclerosis MK-0524-A (tredaptive), MK-0524B, and MK-0859 (anacetrapib).

In summary, treatment and control of arteriosclerotic vascular disease in hypertensive patients needs the overall treatment of the risk factors; in particular, in patients with dyslipemia, it is necessary to normalize blood pressure, to reduce LDL-C values to normal levels by the inhibition of endogenous and exogenous cholesterol, as well as to decrease triglycerides, and increase HDL-C levels, so that it is essential to maintain dietetic measures (reduction of salt and unsaturated fat intakes) and continuous pharmacologic treatment in most of the patients.

## Figures and Tables

**Table 1:** Levels of total cholesterol, triglycerides, HDL cholesterol, and LDL cholesterol according to NECP Guidelines. Adult Panel III (2002).

Cholesterol	Goal
Ideal	<200 mg/dL
High limit	200–239 mg/dL
High	>239 mg/dL
HDL cholesterol	40–55 mg/dL
LDL cholesterol	50–130 mg/dL
Triglycerides	50–150 mg/dL

**Table 2:** Drugs recommended to normalise lipid disorders.

Drugs to reduce LDL cholesterol	New statins
	ACAT inhibitors
	MTP inhibitors
	Bile acids transport inhibitors
	Specific cholesterol absorption inhibitors (Ezetimibe)

Drugs that increase HDL cholesterol	Nicotinic acid
	PPAR *α*/*β* dual agonists
	Lipoprotein lipase activators
	CETP inhibitors

Drugs to treat Hypertriglyceridemia	Fibric acid derivatives
	Icosapent ethyl ester
	Doconexent ethyl ester

ACAT: Acyl-CoA cholesterol acyl transferase; MTP: microsomal triglyceride transfer protein; PPAR: proliferator-activated receptor; CETP: cholesteryl ester transfer protein.

**Table 3:** National Cholesterol Education Program. Therapeutic target according with the lipids blood levels.

Category of risk	Target LDL-Ch	LDL-Ch levels for no drugs	LDL-Ch level for drug use
IC (risk to 10 years >20%)	<100	≥100	>130; 100–129 optional therapy
2+ risk factors, risk to 10 years (≤20%)	<130	≥130	Risk to 10 years 10–20% ≥130 risk 10 years <10% ≥160
0-1 risk factors	<160	≥160	≥190; 160–189 LDL-Ch optional lipid drugs

IC: ischemic coronary disease.
